# The Chicken or the Egg? An Interesting Case Presentation of Spontaneous Coronary Artery Dissection Versus Takotsubo Cardiomyopathy

**DOI:** 10.7759/cureus.7793

**Published:** 2020-04-23

**Authors:** Anjali R Desai, Puja Patel, Raj Patel, Harshavardhan Ghadiam, Ekanka Mukhopadhyay

**Affiliations:** 1 Internal Medicine, University of Illinois College of Medicine at Peoria, Peoria, USA; 2 Internal Medicine, American University of Antigua, Peoria, USA; 3 Cardiology, University of Illinois College of Medicine at Peoria, Peoria, USA

**Keywords:** cardiology research, cardiology imaging, cardiac catheterization, stress induced cardiomyopathy

## Abstract

This is an interesting cardiovascular imaging and coronary angiography case of a 67-year-old female patient who presented with chest pain, abnormal electrocardiogram (EKG), and heart failure who was subsequently found to have spontaneous coronary artery dissection (SCAD) and Takotsubo cardiomyopathy (TCM) on imaging studies. The case presentation highlights the importance of imaging studies and prompt diagnosis in these patients. This study may also highlight the need for early medical intervention in patients with suspected systolic dysfunction due to either of these pathophysiologic processes.

## Introduction

Traditionally considered mutually exclusive where coronary disease needs to be ruled out before the diagnosis of stress-induced cardiomyopathy; Takotsubo cardiomyopathy (TCM) and spontaneous coronary artery dissection (SCAD) share common pathophysiologic mechanisms and may occasionally manifest together [1]. The mainstay of treatment for either condition is generally conservative, with SCAD being treated with usual acute coronary syndrome medications and TCM being treated with guideline-directed heart failure therapies [2]. This case highlights the importance of recognizing that both entities can present in the same setting. Which condition begets the other is a conundrum and not known; however, the treatment modality should include the management of both conditions and prevention of future adrenergic flares (i.e. anxiety, exacerbation of other chronic conditions, etc.) [3].

## Case presentation

**Figure 1:**
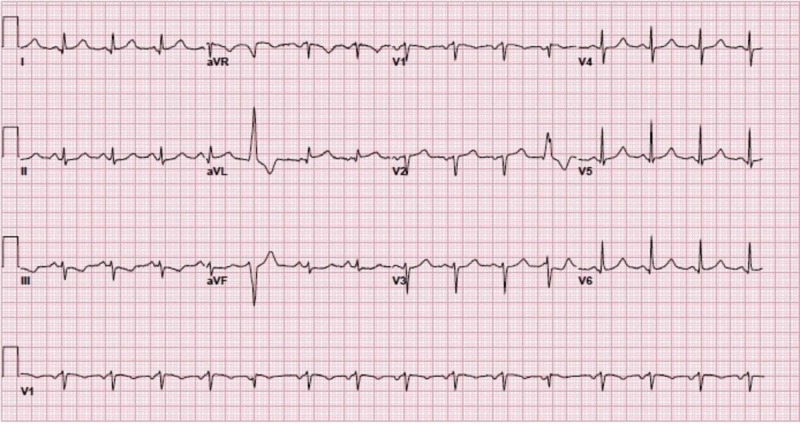
Electrocardiogram on presentation showing lateral ST segment elevation and reciprocal inferior ST segment depression

**Figure 2:**
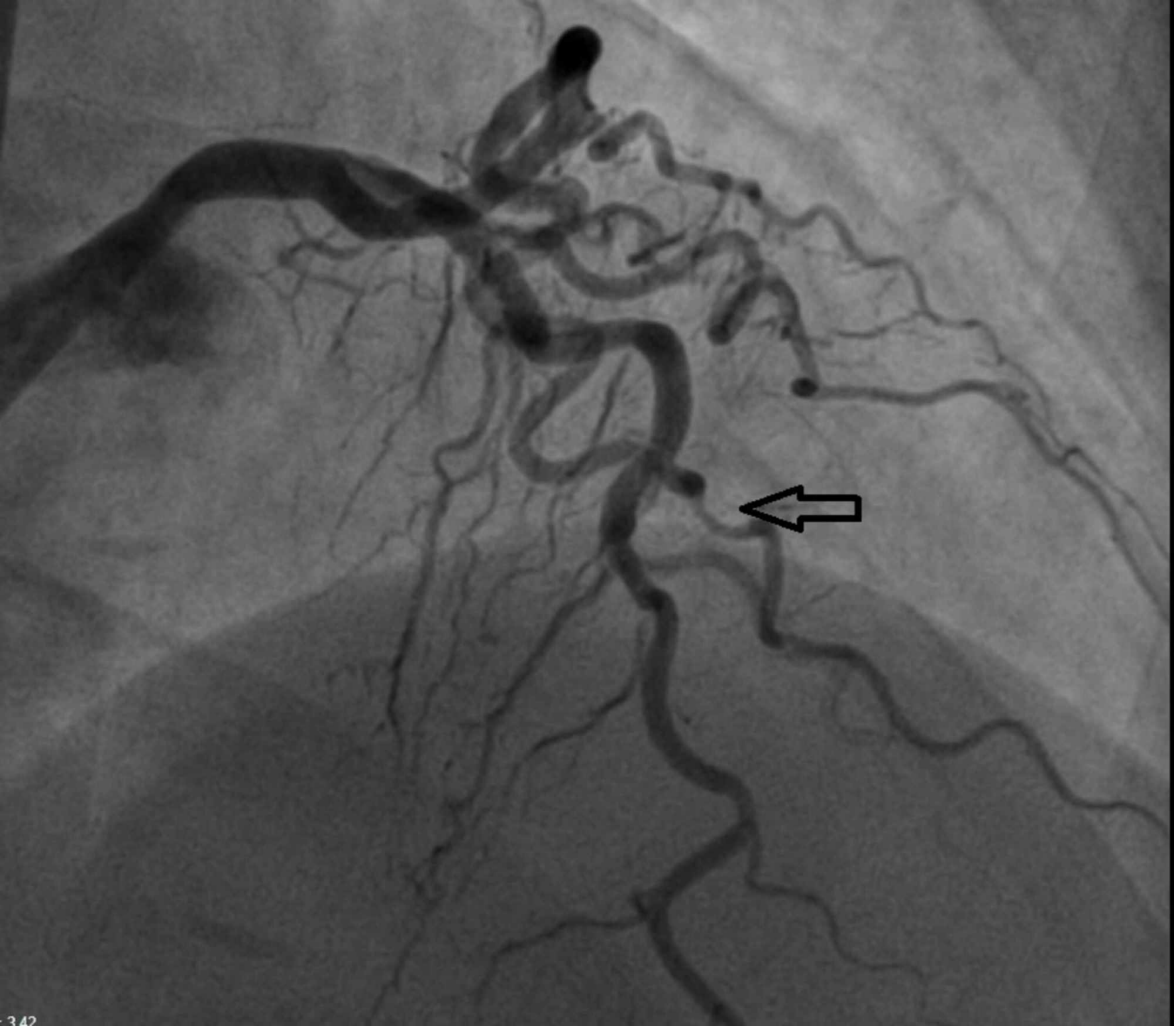
Coronary angiogram revealing evidence of spontaneous coronary artery dissection in the diagonal branch of the left anterior descending artery

We present the case of a 67-year-old Caucasian female with prior tobacco use, essential hypertension, hyperlipidemia, coronary artery disease, status post (s/p) remote percutaneous coronary intervention (PCI) to the right coronary artery who presented to the hospital with complaints of chest discomfort and shortness of breath. The patient was found to have acute lateral ST segment elevations on the presenting electrocardiogram (EKG) (Figure 1). The patient was treated for acute coronary syndrome and urgently taken to the cardiac catheterization laboratory where she underwent left heart cardiac catheterization and coronary angiography. Angiogram revealed the previously placed mid right coronary artery stent to be widely patent. The left circumflex and main left anterior descending arteries showed minimal luminal irregularities. However, the second diagonal branch of the left anterior descending artery did reveal a dissection plane (Figure 2). Due to small caliber vessel, no further intervention was attempted. The coronary angiogram was followed by ventriculography which revealed a typical TCM pattern of the left ventricle with hyperkinesis of the basal segments and akinesis/ballooning of the mid and apical segments (Video 1). The patient was managed medically for SCAD and given guideline directed medical therapy for stress induced cardiomyopathy.

**Video 1:** Ventriculography showing basal segment hyperkinesis with mid to apical ballooning typical of stress-induced cardiomyopathy

## Discussion

TCM and SCAD have underlying commonalities. They are both non-atherosclerotic causes of myocardial infarction and patients typically present with ACS like features; i.e. chest discomfort, cardiac enzyme elevation, and EKG changes to suggest ischemia [1]. Both conditions share female predominance [1]. The underlying pathophysiology is likely associated with sympathetic surges related to emotionally taxing or stressful events [2,3].

There are stark differences in the two conditions as well. For example, while SCAD generally presents with wall motion abnormalities specific to the coronary artery affected, TCM may have the typical "Octopus sac" appearance with basal segment hyperkinesis and apical ballooning of the left ventricle [4-6]. 

When the conditions manifest concurrently, it is unclear whether SCAD begets TCM or if TCM begets SCAD. There have been mechanisms postulated for both mechanisms. Firstly, the initial pain, anxiety, and discomfort associated with SCAD may lead to an additional adrenaline surge which furthermore increases the predisposition to stress-induced cardiomyopathy [4]. If the primary insult is TCM, the torsional and stretch forces associated with basilar hyperkinesis and apical ballooning may be sufficient enough to lead to intimal tears in the epicardial coronary arteries predisposing the patient to SCAD [1]. Irrespective of the postulated mechanism, it is clear, as demonstrated in our patient, that these two conditions can and do occur simultaneously. 

Other medical centers have reported similar cases of TCM and SCAD presenting concurrently. For instance, Ghafoor et al. reported the case of TCM/SCAD with similar clinical findings as our patient. They reported commonality in the pathophysiology in addition to considering early institution of heart failure management in patients that are presumed to have concurrent TCM with SCAD [7]. 

It is not entirely clear as to what degree heart failure will manifest in SCAD patients, if at all. However, as there is a clear association in some patients with TCM and SCAD, early initiation of beta-blocker and angiotensin-converting enzyme (ACE) inhibitor therapy may be considered when either diagnosis is suspected. Further studies should be done on long-term heart failure therapy and the implication on future flares of TCM, SCAD, or combined events.

## Conclusions

TCM and SCAD are two distinct pathological processes that share common pathophysiologic mechanisms. It is imperative to recognize that these processes may manifest together and treatment should be guided by underlying clinical presentation. Early initiation of ACE inhibitors and beta blockers may be warranted in certain patients with these pathologic entities. Further studies should be done to evaluate specific characteristics and subsets of patients who may have a predilection for developing both TCM/SCAD concurrently.
